# Impact of COVID-19 on the Epidemiological Features of Mycoplasma pneumoniae Infection in Children with Community-Acquired Pneumonia in Henan, China

**DOI:** 10.1128/spectrum.04911-22

**Published:** 2023-01-23

**Authors:** Lifeng Li, Pengbo Guo, Jiayue Ma, Huiqing Sun, Shiyue Mei

**Affiliations:** a Henan International Joint Laboratory of Children’s Infectious Diseases, Children’s Hospital Affiliated to Zhengzhou University, Henan Children’s Hospital, Zhengzhou Children’s Hospital, Zhengzhou, China; b Department of Neonatology, Children’s Hospital Affiliated to Zhengzhou University, Henan Children’s Hospital, Zhengzhou Children’s Hospital, Zhengzhou, China; University of Mississippi Medical Center

**Keywords:** COVID-19, *Mycoplasma pneumoniae*, community-acquired pneumonia, epidemiological features

## LETTER

Mycoplasma pneumoniae is an atypical pathogen that can cause respiratory tract and extrapulmonary infections, the most common of which is community-acquired pneumonia (CAP) (1). It typically accounts for 10% to 40% of pneumonic pathogens in children and adolescents, whereas the proportion increased to 20% to 70% during the epidemic period (2). Coinfection with severe acute respiratory syndrome coronavirus 2 (SARS-CoV-2) and M. pneumoniae was also reported; the proportion of cases may have reached 26% (3). Nonpharmaceutical interventions such as social distancing and mask wearing were taken to prevent the transmission of SARS-CoV-2, which may also prevent the transmission of other respiratory tract pathogens (4). Hence, epidemiological monitoring is required to reveal the prevalence of M. pneumoniae infection.

Chen et al. published their results in *Microbiology Spectrum*, reporting the prevalence of M. pneumoniae infection in children with mild respiratory tract infections from January 2020 to June 2021 (5). Sauteur et al. reported a decline in M. pneumoniae infections during the coronavirus disease 2019 (COVID-19) pandemic (6). To provide further insights, we analyzed the prevalence of M. pneumoniae infection based on laboratory detection in children with CAP before and during the COVID-19 pandemic in Henan, China.

**FIG 1:**
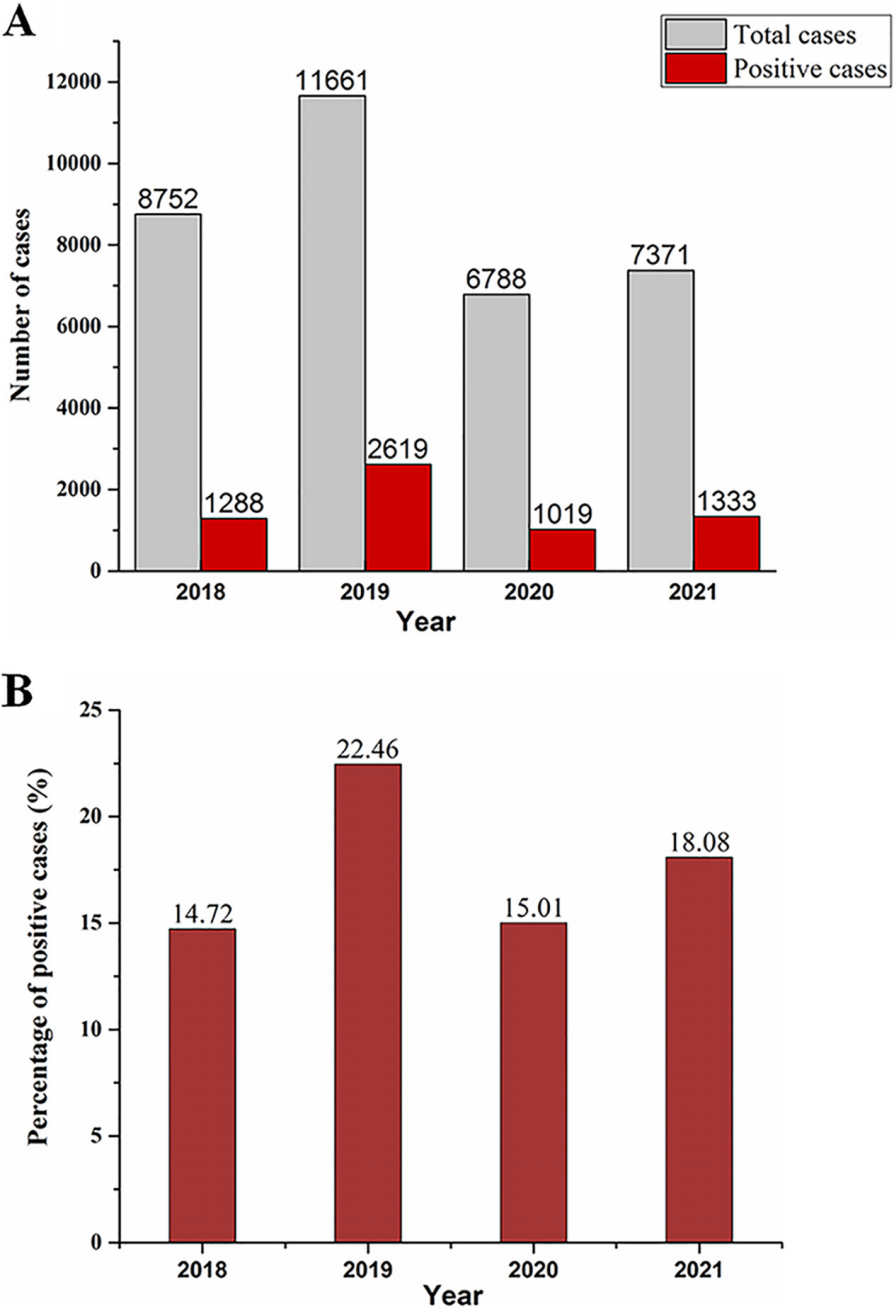
Number of cases (A) and positivity rates (B) of M. pneumoniae infection in children with CAP from 2018 to 2021.

Data were collected at Henan Children’s Hospital from January 2018 to December 2021. Reverse transcription-PCR (RT-PCR) was used to detect M. pneumoniae (2). In total, 34,752 CAP-infected children were included (*n* = 8,752 in 2018, *n* = 11,661 in 2019, *n* = 6,788 in 2020, *n* = 7,371 in 2020) (Fig. 1A); the total proportion of positive cases was 18.10%. Before COVID-19, the proportion of positive cases was 14.72% (1,288/8,752) in 2018 and 22.46% (2,619/11,661) in 2019. During the course of the COVID-19 pandemic, the proportion of positive cases was 15.01% (1,019/6,788) in 2020 and 18.08% (1,333/7,371) in 2021. A decreased positive rate was observed in 2020, but it increased again in 2021 (Fig. 1B).

**FIG 2:**
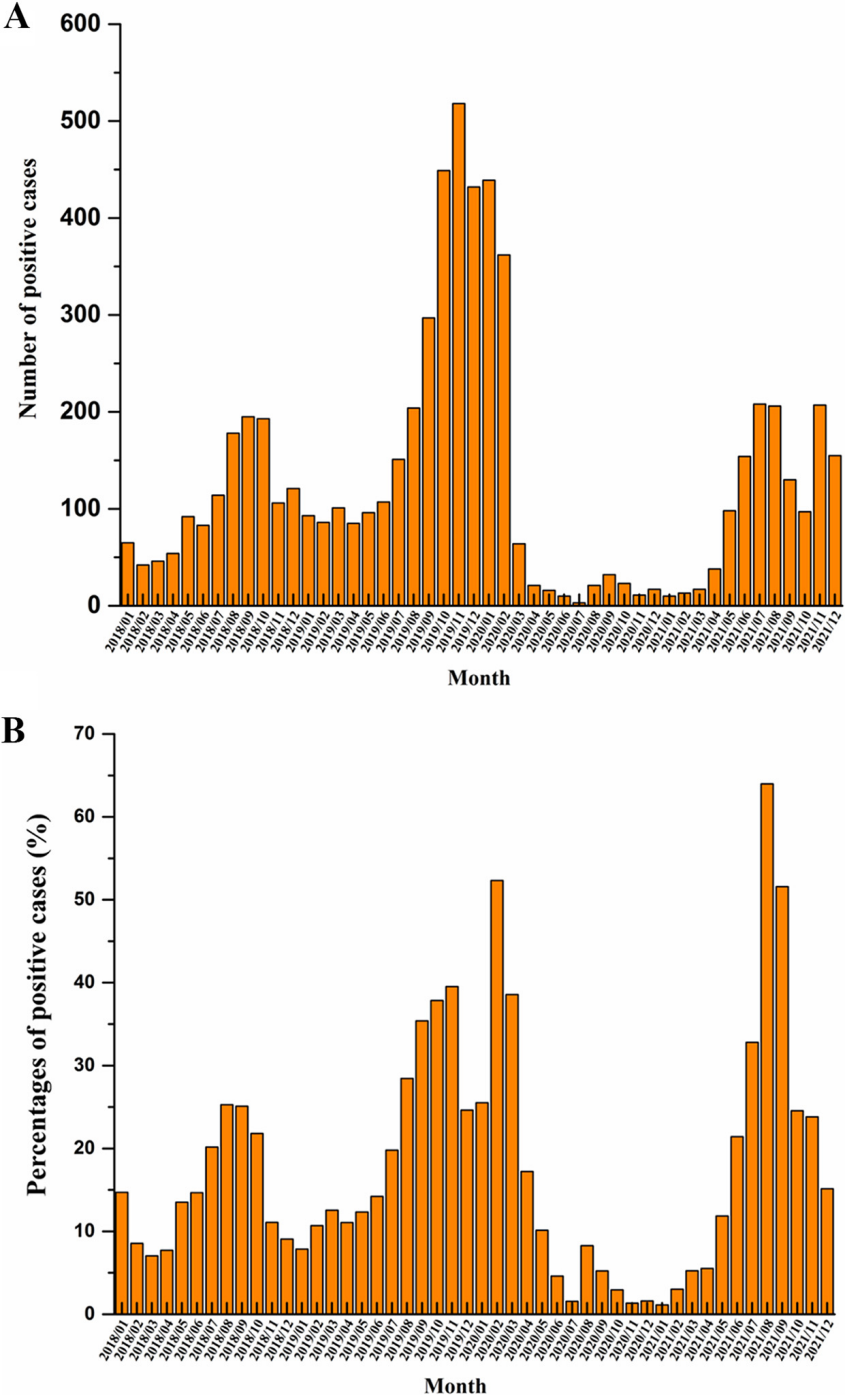
Number of positive cases (A) and positivity rates (B) of M. pneumoniae infection in children with CAP per month from 2018 to 2021.

We also analyzed M. pneumoniae infections by month and found that they were not evenly distributed (Fig. 2). The month with the highest number of positive cases in 2018 was September; in 2019, it was November, in 2020, January, and July in 2021. The overall number of positive cases was lowest in 2020, which indicated the effect of COVID-19 preventative measures such as social distancing, online learning, and mask wearing. Higher prevalence rates were found from July to November (summer and autumn) (from 20.18% to 39.54%) in 2018 and 2019 before COVID-19, whereas the positive rates were higher from January to April (winter and spring) in 2020 (from 17.21% to 52.31%) (Fig. 2B). The proportion of positive cases from May to December in 2020 was lower than that in the other 3 years (from 1.34% to 15.01%) due to strict COVID-19 interventions. Surprisingly, the months with the highest proportion of positive cases returned to July to October in 2021 (from 24.56% to 63.96%), which may be related to the withdrawal of restrictions and preventive measures adopted for COVID-19. The highest proportion of positive cases was 25.28% in August 2018 and 39.54% in November 2019, reaching 52.31% in February 2020 and 63.96% in August 2021 during COVID-19.

In summary, we reported here the fluctuating prevalence of M. pneumoniae infection in children with CAP and changes in the seasonal distribution from 2018 to 2021. Extremely high monthly proportions of positive cases were reported in 2020 and 2021. Continuous monitoring of M. pneumoniae infections will be helpful for the prevention and control of related infections.

### Data availability.

The data are available from the corresponding authors upon request.
